# Quantitative Ultra-Performance Liquid Chromatography Tandem Mass Spectrometry Method for Comparison of Prochloraz Residue on Garlic Sprouts after Soaking and Spraying Treatment

**DOI:** 10.3390/ijerph15071552

**Published:** 2018-07-23

**Authors:** Qingkui Fang, Chenchun Ding, Zhan Dong, Shuai Guan, Ruifeng Wu, Xiangwei Wu, Rimao Hua, Haiqun Cao

**Affiliations:** 1 School of Plant Protection, Provincial Key Laboratory for Agri-Food Safety, Anhui Agricultural University, Hefei 230036, China; qkfang@163.com; 2 School of Resource & Environment, Provincial Key Laboratory for Agri-Food Safety, Anhui Agricultural University, Hefei 230036, China; chenchundingdcc@163.com (C.D.); 15056960209@163.com (R.W.); wxw@ahau.edu.cn (X.W.); rimaohua@ahau.edu.cn (R.H.); 3 Institute of Agricultural Quality Standards and Testing Technology, Shandong Academy of Agricultural Sciences, Ji’nan 250100, China; dong_zhan@126.com (Z.D.); guanshuai831726@163.com (S.G.)

**Keywords:** Garlic sprout, Prochloraz, ultra-performance liquid chromatography tandem mass spectrometry, pesticide residue

## Abstract

Prochloraz is a fungicide that is widely used on vegetables to maintain freshness during storage. To ensure that prochloraz is used in a safe way that reduces the levels of residue on the product, we evaluated two treatment methods (soaking and spraying) that are commonly used for garlic sprouts. An ultra-performance liquid chromatography tandem mass spectrometry (UPLC-MS/MS) method was developed and validated for prochloraz residue on garlic sprouts. The linear range of the method was 5–500 μg/kg and the correlation coefficient was 0.9983. The average recovery range was 88–94%, and the relative standard deviation range was 2.6–9.7%. Garlic sprout samples that had been soaked in or sprayed with prochloraz were collected from cold storage facilities in Laixi and Pingdu, China. For the soaked samples, the ranges for the levels of prochloraz residue on the whole garlic sprouts and stems (edible portion) were 15.76–25.14 mg/kg and 0.58–1.62 mg/kg, respectively. For the sprayed samples, the ranges for the levels of prochloraz residue on the whole garlic sprouts and stems were 1.85–7.89 mg/kg and 0.01–1.29 mg/kg, respectively. The results of this study provide a scientific basis for rationalizing the use of prochloraz and improving the safety of edible garlic sprouts.

## 1. Introduction

Garlic sprouts are a common and popular vegetable in north China [1]. They are rich in allicin, sugar, crude fiber, carotene, vitamin A, vitamin B_2_, vitamin C, niacin, calcium, phosphorus, and other nutrients [2]. The bactericidal ability of allicin can reach one-tenth of that of penicillin [3] and garlic sprouts can help prevent influenza and wound infection, as well as be used for deworming [4].

Of the types of vegetables kept in cold storage facilities in China, garlic sprouts make up the largest proportion and are stored for longer periods than other vegetables [5]. Garlic sprouts are generally harvested in May [6], when the average temperature in Shandong Province (China) is high, with temperatures recorded in Jinan of 22.5 °C in 2015, 20.9 °C in 2016, and 24.0 °C in 2017 (http://tianqi.2345.com/wea_history/54823.htm). In these temperatures, garlic sprouts can lose water, leading to aging and decay [7,8,9,10]. In addition, flower bud growth is strong under these conditions, and if garlic sprouts are not stored under low temperature, high humidity, and low oxygen conditions, stem nutrients and water will quickly transfer to the sprouts [11,12,13,14], leading to aging and fibrosis. However, if the humidity is too high, mold will grow on the garlic sprouts, and in severe cases this may even lead to rot. Consequently, fungicides are often applied to garlic sprouts before storage [15,16]. 

Prochloraz is a broad-spectrum imidazole fungicide with high efficiency and low toxicity. However, at the same time, prochloraz has been suspected of acting as an endocrine disrupter [17,18]. It is widely used on fruits and vegetables to prevent deterioration during storage [19,20]. However, prochloraz residue on agricultural products may pose a risk to human health and environmental safety [21,22]. It is generally known that the scientific use of pesticides and accurate assessment of pesticide residue can reduce unnecessary exposure, which in turn could benefit both humans and the environment. 

To study the scientific use of prochloraz and undertake accurate assessments of prochloraz residue on garlic sprouts, in this study, we developed an ultra-performance liquid chromatography tandem mass spectrometry (UPLC-MS/MS) method for detecting prochloraz residue on garlic sprouts. We compared the levels of prochloraz residue on garlic sprouts after spraying and soaking treatments, and evaluated the health risk of prochloraz residue on garlic sprouts. 

## 2. Materials and Methods

### 2.1. Reagents

Stock solutions (1000 mg/L) of prochloraz, for the preparation of prochloraz solutions of different concentration gradients, were stored in capped glass vials in the dark at 2 °C until required for use. A working solution (50 μg/kg) for calibration was prepared by diluting the stock solutions. Petroleum ether and acetone were obtained from Pharmco Products Inc. (Brookfield, CT, USA) and acetonitrile was obtained from Sigma-Aldrich (Steinheim, Germany). Sodium chloride was purchased from J. T. Mallinckrodt Baker Inc. (Phillipsburg, NJ, USA).

### 2.2. Postharvest Treatment and Storage

Garlic sprout samples that had been treated with prochloraz were collected from cold storage facilities in Shandong Province (Laixi and Pingdu, China). The garlic sprouts were treated by either soaking or spraying, and we collected two sprayed samples and two soaked samples from each of four cold storage facilities. A prochloraz aqueous suspension was prepared by diluting a commercial formulation of 25% prochloraz emulsifiable concentrate (Qingdao Star Cropscience Co., Ltd., Qingdao, China) with tap water. All garlic samples were treated with a prochloraz solution containing 500 mg of active ingredient per liter. Soaking treatments were carried out by dipping the flower buds of the garlic sprouts in the prochloraz aqueous suspension for 2 min. Spraying treatments were carried out by spraying the flower buds of the garlic sprouts with the prochloraz aqueous suspension. All treated samples were dried in a ventilated area and then stored at 2 °C in a cold storage facility. All samples were treated with prochloraz on 22 May 2016. Samples (50 g each) for residue analysis were collected on 1 June, 29 June, and 30 July 2016. Because the fungicide was mainly applied to the flower buds of the garlic sprouts, the samples used for residue analysis were divided into whole plant samples and stem (edible portion) samples without the flower buds.

### 2.3. Sample Preparation

#### 2.3.1. Sample Extraction

The garlic sprouts samples were finely chopped and homogenized. The homogenized garlic sprout samples were weighed into 100 mL centrifuge tubes (5.0 g per tube), then 20 mL of acetonitrile was added to each tube and the tubes were thoroughly shaken for 1 min. Next, 5.0 g of NaCl was added to each tube, and the tubes were ultrasonicated for 15 min and then centrifuged at 4000× *g* for 5 min. An aliquot (10 mL) of the supernatant was removed from each centrifuge tube and placed in a 150 mL flask. An additional 20 mL of acetonitrile was added to each 100 mL centrifuge tube, and the above extraction steps with NaCl were repeated. A 10 mL aliquot of the supernatant from the second extraction for each sample was transferred to the corresponding 150 mL flask. The combined extracts for each sample were concentrated close to dryness at 40 °C.

#### 2.3.2. Purification

A NH_2_ solid-phase extraction (SPE) column was pre-leached with 5 mL of petroleum ether:acetone (7:3, *v/v*). Next, two 3 mL portions of petroleum ether:acetone (7:3, *v/v*) were added to each sample in a flask. The upper liquid was removed and added to the SPE column, the SPE column was rinsed with 5 mL of petroleum ether:acetone (7:3, *v/v*), and the eluate was discarded. Then, the SPE column was rinsed with acetonitrile (3 × 5 mL), and the eluate was collected into a flask and concentrated close to dryness at 40 °C. The volume was set to 5 mL and placed in a volumetric flask with acetonitrile:water (3:2, *v/v*) to await measurement.

#### 2.3.3. Method Validation

Calibration curves and garlic sprout samples were prepared using working standard solutions. Samples that were verified by UPLC-MS/MS to not contain prochloraz were used as blank samples. The blank samples were spiked with 5, 50, and 500 μg/kg of prochloraz, and subjected to the UPLC-MS/MS analysis to evaluate the accuracy and precision of the method. The slopes of the calibration curves for the samples prepared in solvent and in the matrix were compared. To minimize matrix effects, the linearity was studied using calibration curves prepared with matrix-matched standards. The limit of quantification (LOQ, 5 μg/kg) was determined as the lowest concentration meeting the method performance criteria for trueness and precision for a given compound. Recovery tests were repeated five times for each spiking level.

### 2.4. UPLC-MS/MS Conditions

The MS/MS transition was undertaken according to an established method [23], and the LC-MS/MS methods were prepared as described previously [23,24], with slight modification. An Acquity UPLC equipped with a BEH (ethylene bridged hybrid) C18 column (100 mm × 2.1 mm, 1.7 µm particle size; Waters, Milford, MA, USA) was coupled to a Xevo TQ triple quadruple mass spectrometer (Waters) and operated in the positive electrospray ionization mode for UPLC-MS/MS. The LC was operated under gradient conditions with mobile phases of A (water/methanol (98:2, *v/v*) + 0.05% formic acid) and B (methanol + 0.05% formic acid) at 40 °C. The mobile phase flow rate was 0.45 mL/min. A gradient elution was performed as follows: 0–0.25 min, 5% B; 0.25–8.50 min, 100% B and 8.50–10 min, 5% B. The total run time was 10 min. The sample injection volume was 3 µL.

The mass spectrometry source temperature was 150 °C, and the nitrogen gas flow rates for the cone and desolvation gases were 50 and 800 L/h, respectively. The desolvation temperature was 500 °C. Argon was used as the collision gas with a flow rate of 0.15 mL/min. The mass spectrometer was operated in the multiple reactions monitoring mode, and two precursor/product ion transitions were monitored for each analyte. The target ion transition with the highest intensity (primary ion transition) was used for quantitation, and the second target ion transition was used for confirmation. Further confirmation was obtained through a product ion scan for each peak, which was matched to a reference spectrum for each analyte. The quantification and confirmation calculations were performed using the software Target Lynx 4.1 (Waters Corp, Milford, MA, USA). Ion transitions, cone voltages, collision energies, and dwell times for the analytes are shown in Table 1.

## 3. Results and Discussion

### 3.1. Limit of Detection and LOQ

The limit of detection (LOD) was calculated as three times the value of the instrument background signal generated by the blank matrix, and the LOQ was calculated as 10 times the background signal generated by the blank matrix (Table 2). The ion ratio was established using the corresponding ratio of a standard. The LOD and LOQ for prochloraz were 0.0166 μg/kg and 0.0499 μg/kg, respectively. In practical experiments, the minimum calibration level for the samples was set at 5 μg/kg, because of matrix interference.

### 3.2. Linearity

Linearity was evaluated using blank extracts at seven concentration levels (5, 10, 20, 50, 100, 200, and 500 μg/kg) (Table 2). For quantification, calibration was carried out using external standards, and the correlation coefficient was 0.9983. These results show the method has good linearity, and is suitable for prochloraz detection. 

### 3.3. Recovery Study

In the chemical analysis, the matrix refers to components other than the analyte in the sample. The matrix can greatly interfere with analysis of the analyte, and the accuracy of the analytical results. There are many ways to eliminate matrix effects, such as the removal of matrix components by purification, special injection methods, standard addition, or the use of a stable-isotope labeled internal standard or analyte protectant [21,25,26]. One of the aims of this research was to apply a method to garlic sprout samples to determine the levels of prochloraz residue on the garlic sprouts. Matrix effects are commonly removed using calibration curves prepared with standard samples of known analyte concentrations, while keeping the matrix in the sample as constant as possible. The analyte recoveries and precision values obtained from the validation study are summarized in Table 3. The mean recovery range for prochloraz was 88.4–94.8%, with a standard deviation (SD) and relative standard deviation range of 0.46–11.69 μg/kg and 2.6–9.7%, respectively. For prochloraz, the matrix effect was 5.8%. Furthermore, we repeated the measurement recovery (5, 50, and 500 μg/kg) three times for the inter-day. The inter-assays’ relative standard deviations (RSDs) were 9.8%, 6.2% and 5.9%, respectively. This clearly indicated the good reproducibility of this UPLC-MS/MS approach.

### 3.4. Actual Sample

**Figure 1:**
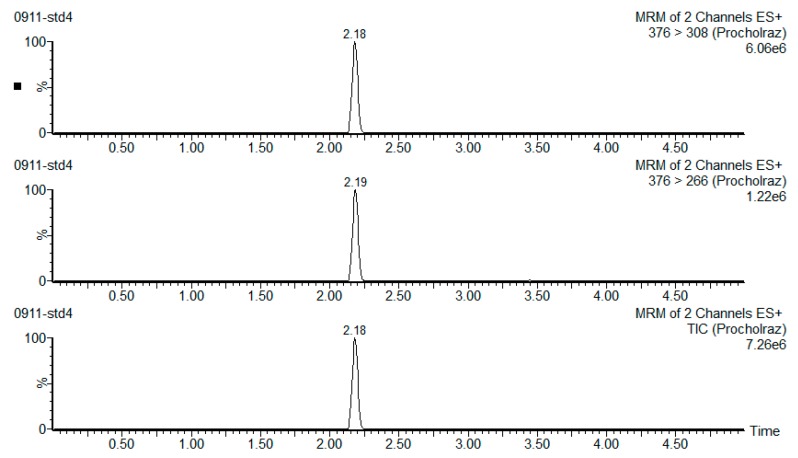
An example of an extracted multiple reaction monitoring mode (MRM) chromatogram of a garlic sprout sample, indicating the presence of prochloraz.

Prochloraz is widely used in agriculture to maintain vegetable and fruit freshness. To rationalize the use of prochloraz in cold storage and evaluate the safety of edible garlic sprouts, we determined the levels of residue of prochloraz on garlic sprouts (Table 4). A gradual deterioration of prochloraz residue in the two treated garlic sprout samples was observed during the storage process. We looked at differences in the levels of prochloraz residue after soaking and spraying treatments, and differences between the whole plant and the stems (edible portion). After soaking treatment, the range for the level of prochloraz residue on the whole plant was 21.86–25.14 mg/kg for the samples collected in Pingdu, and 15.76–20.18 mg/kg for the samples collected in Laixi. The levels of prochloraz residue on the stems were 0.58–1.12 mg/kg for the Pingdu samples, and 1.04–1.62 mg/kg for the Laixi samples. After spraying, the range for the level of prochloraz residue on the whole plant was 1.85–5.93 mg/kg for the Pingdu samples, and 4.08–7.89 mg/kg for the Laixi samples. For the stem samples, the results were 0.01–1.11 mg/kg for the Pingdu samples and 0.03–1.29 mg/kg for the Laixi samples. An emblematical chromatogram of a garlic sprout sample is exhibited in Figure 1.

These results show that the levels of prochloraz residue present on the whole plant after spraying (1.85–7.89 mg/kg) are lower than those after soaking (15.76–25.14 mg/kg). Therefore, the use of spraying over soaking for treatment during storage could reduce the levels of prochloraz residue on garlic sprouts. Furthermore, lower prochloraz residue levels were found for the stem samples than the whole plant samples for both soaking (stem: 0.58–1.62 mg/kg; whole plant: 15.76–25.14 mg/kg) and spraying (stem: 0.01–1.29 mg/kg; whole plant: 1.85–7.89 mg/kg). To date, a maximum residue limit has not been established for prochloraz on garlic sprouts. However, according to the European Union Pesticides database, the maximum residue limit for prochloraz on garlic is 0.5 mg/kg, and that for chives or shallots is 5.0 mg/kg. In addition, the maximum residue limit for prochloraz on flowering Chinese cabbage is 2.0 mg/kg, as established by Chinese regulation. Therefore, we suggest that the buds of garlic sprouts are removed before consumption to reduce the intake of prochloraz residue.

## 4. Conclusions

In this study, we developed a rapid and efficient UPLC-MS/MS method to determine the levels of prochloraz residue on garlic sprouts. A comparison of garlic sprouts treated by spraying and soaking showed that the levels of prochloraz residue were lower after spraying than after soaking. Prochloraz residue levels for the stems were lower than those for the whole plant. Therefore, to reduce the intake of prochloraz by humans and for environmental safety, we recommend the spraying method is used for prochloraz application during storage, and that the flower buds of the garlic sprouts are not eaten. 

## Figures and Tables

**Table 1:** Ion transitions used for quantification (multiple reaction monitoring modes (MRM) 1) and confirmation (MRM2), dwell time, cone voltages, and collision energies for MS.

Compound	Transitions	Dwell Time (s)	Cone Voltage (V)	Collision Energy (eV)
Prochloraz	Quantification ion: 376 > 308	0.008	20	15
	Confirmation ion: 376 > 266		20	15

**Table 2:** Matrix effects (ME), retention time (*t*_R_), linear range, linear regression equation, and linearity.

Pesticide	ME (%)	*t*_R_ (min)	Linear Range (μg/kg)	Linear Regression Equation	Linearity
Prochloraz	5.8	2.20	5–500	Y = 0.9898X − 1.2624	0.9983

**Table 3:** UPLC-MS/MS recovery studies of samples spiked with prochloraz (*n* = 5).

Pesticide	LOD (μg/kg)	LOQ (μg/kg)	Concentration (μg/kg)	Measured ± SD (μg/kg)	Recovery (%)	RSD (%)
Prochloraz	0.0166	0.0499	5	4.7 ± 0.46	94.8	9.7
			50	44.2 ± 1.26	88.4	2.8
			500	447.1 ± 11.69	89.4	2.6

**Table 4:** Actual samples of prochloraz residue after soaking and spraying treatment (*n* = 3).

Sampling City	Sampling Time	Soaking (mg/kg)		Spraying (mg/kg)	
		Whole Plant (Measured ± SD)	Stems (Measured ± SD)	Whole Plant (Measured ± SD)	Stems (Measured ± SD)
Pingdu	1 June	25.14 ± 1.20	1.12 ± 0.07	5.93 ± 0.36	1.11 ± 0.06
	29 June	22.72 ± 0.94	0.80 ± 0.03	2.23 ± 0.18	0.12 ± 0.01
	30 July	21.86 ± 1.13	0.58 ± 0.04	1.85 ± 0.12	0.01 ± 0.001
Laixi	1 June	20.12 ± 0.86	1.62 ± 0.05	7.89 ± 0.54	1.29 ± 0.09
	29 June	18.70 ± 0.42	1.13 ± 0.09	5.04 ± 0.30	0.15 ± 0.01
	30 July	15.76 ± 1.06	1.04 ± 0.06	4.08 ± 0.20	0.03 ± 0.003
